# QuickStats

**Published:** 2014-12-12

**Authors:** FROM THE NATIONAL CENTER FOR HEALTH STATISTICS

**Figure:**
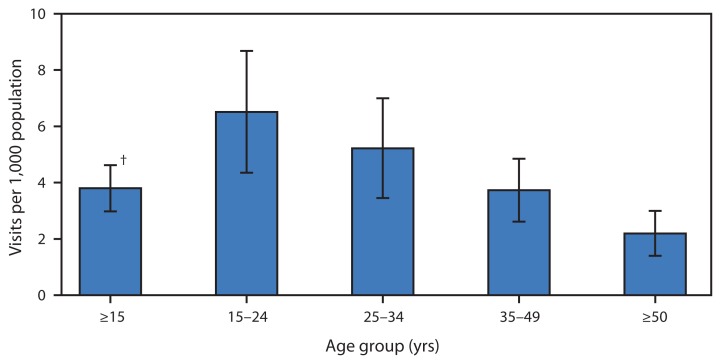
Average Annual Rate of Emergency Department Visits for Bipolar Disorder^*^ Among Persons Aged ≥15 Years, by Age Group — National Hospital Ambulatory Medical Care Survey, United States, 2010–2011 ^*^ Per 1,000 population based on the annual average over 2 years. Visits for bipolar disorder were defined as those with any of the following *International Classification of Diseases, Ninth Revision*, *Clinical Modification* diagnosis codes: 296.0, 296.1, or 296.4–296.8. Up to three diagnoses were recorded for each visit. ^†^ 95% confidence interval.

During 2010–2011, approximately 468,000 emergency department visits were made each year by persons aged ≥15 years with a diagnosis of bipolar disorder, an overall rate of 3.8 visits per 1,000 persons per year. The visit rate declined significantly as age increased. Persons aged 15–24 years had the highest rate (6.5 per 1,000), which was nearly three times the rate for persons aged ≥50 years (2.2 per 1,000).

**Source:** National Hospital Ambulatory Medical Care Survey, 2010–2011. Available at http://www.cdc.gov/nchs/ahcd.htm.

**Reported by:** Donald K. Cherry, MS, dcherry@cdc.gov, 301-458-4762; Linda F. McCaig, MPH.

